# Small bowel intussusception from renal cell carcinoma metastasis: a case report and review of the literature

**DOI:** 10.1186/s13256-016-0998-0

**Published:** 2016-08-11

**Authors:** Gabriele Bellio, Tommaso Cipolat Mis, Gladiola Kaso, Roberto Dattola, Biagio Casagranda, Marina Bortul

**Affiliations:** 1 Department of General Surgery, Cattinara University Hospital, Trieste, Italy; 2 Department of Radiology, Cattinara University Hospital, Trieste, Italy; 3 Department of Anesthesiology and Critical Care, Cattinara University Hospital, Trieste, Italy

**Keywords:** Intussusception, Renal cell carcinoma, Small bowel, Intestinal occlusion, Ultrasonography, Metastasis

## Abstract

**Background:**

Renal cell carcinoma is the most frequent malignant neoplasia of the kidney accounting for 90 % of all renal solid tumors. Metastases from renal cell carcinoma are rarely located in the small bowel and generally their clinical presentation includes bleeding and obstruction. Intussusception in adults is an extremely rare pathological condition and only 30 to 35 % of small bowel intussusceptions are derived from malignant lesions.

**Case presentation:**

We report here a clinical case of a 75-year-old white man hospitalized for anemia and subocclusion. An abdominal ultrasound and computed tomography showed a small bowel intussusception. During a surgical exploration, a polypoid lesion was found to be the lead point of the intussusception. His small intestine was resected and a functional side-to-side anastomosis was performed. The histological features of the surgical specimen confirmed the diagnosis of metastatic renal cell carcinoma.

**Conclusions:**

Small bowel intussusception from renal cell carcinoma metastasis should always be considered in the setting of unexplained intestinal subocclusion in patients with a history of renal cell carcinoma.

## Background

Intussusception is defined as telescopic penetration of a proximal segment of the gastrointestinal (GI) tract, called “intussusceptum”, into the lumen of the adjacent distal segment of the GI tract, called “intussuscipiens” [1]. Adult intussusception is an extremely rare condition, representing only 5 % of all cases of intussusception [1, 2]. Of all adult intussusceptions, 90 % are secondary to specific and well-known pathologic conditions [1, 2], 65 to 70 % of which are represented by malignancies [1, 3]. This percentage decreases to 30 to 35 % if only small bowel intussusceptions are considered [3]. It is known that intussusceptions due to intraluminal or extraluminal lesions are secondary to the alteration of the normal peristaltic activity caused by those lesions, serving as a lead point [4, 5].

The clinical presentation of adult intussusception is extremely variable and nonspecific. The classic pediatric presentation, represented by the triad of cramping abdominal pain, palpable abdominal mass, and bloody mucoid stool, is rare in adults [6, 7]. We report a case of small bowel intussusception induced by a metastasis from renal cell carcinoma (RCC) surgically treated more than 15 years ago.

## Case presentation

A 75-year-old white man with a history of several admissions to our Emergency Room for recurrent episodes of intestinal subocclusion, nausea, and vomiting, presented to our hospital in April 2015 with melena and anemia. He had a history of left RCC with metastasis in both adrenal glands and his brain. He underwent left nephrectomy and ipsilateral adrenalectomy in 1999. A histopathologic examination revealed a pT1bN0 clear cell RCC with a metastatic lesion in his ipsilateral adrenal gland. In 2004 he was subjected to right adrenalectomy for metachronous metastasis. In 2005 he underwent cerebral radiotherapy for brain metastases from the RCC. He achieved a complete response to radiotherapy, but developed a degenerative encephalopathy with cognitive impairment. After 2 years, in good clinical condition, he decided autonomously not to continue the follow-up. He underwent his last abdominal computed tomography (CT) scan in 2014, with a disease-free result.

**Fig. 1:**
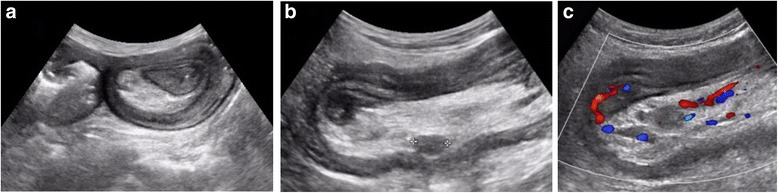
Ultrasonography demonstrated an ileoileal intussusception with the classic “target” sign in the transverse view (**a**) and showed the typical “pseudo-kidney” sign in the longitudinal view with mesenteric lymph nodes within (**b**). The color Doppler highlighted the mesenteric vessels within the intestinal lumen (**c**)

At admission he showed the result of the fecal occult blood test he took at home, which was positive. Blood tests were performed, revealing the known anemia (hemoglobin 7.7 g/dL) and an increase in C-reactive protein (33.9 mg/L). His carcinoembryonic antigen and CA 19.9 were both not beyond the normal values (respectively, 3.6 ng/mL and <0.8 UI/mL). He was then transfused with two blood units because of his low hemoglobin level. An abdominal examination revealed a tender abdominal mass in his right iliac fossa. He did not complain of pain or nausea and he did not have vomiting. He was subjected to gastroscopy and colonoscopy because of the evidence of GI bleeding; the results were negative for GI lesions and active bleeding. An abdominal ultrasonography (US) was performed and it showed a distended bowel loop with thick wall and “target-shaped” feature in the transverse view with two lymph nodes and mesenteric vessels within. These images suggested the presence of bowel intussusception (Fig. 1).

**Fig. 2:**
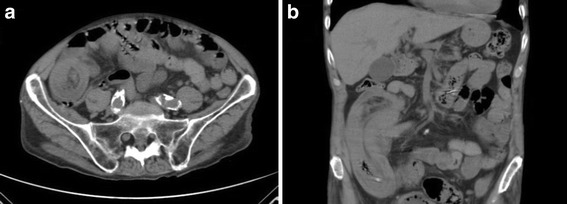
Abdominal computed tomography showed the characteristic inhomogeneous “target-shaped” soft tissue mass with a layering effect in the axial plane (**a**) and the typical “sausage-shaped” soft tissue mass in the coronal plane (**b**)

To better define this diagnosis and to evaluate the extension of the bowel involved, an abdominal CT scan was performed, confirming the US findings (Fig. 2).

**Fig. 3:**
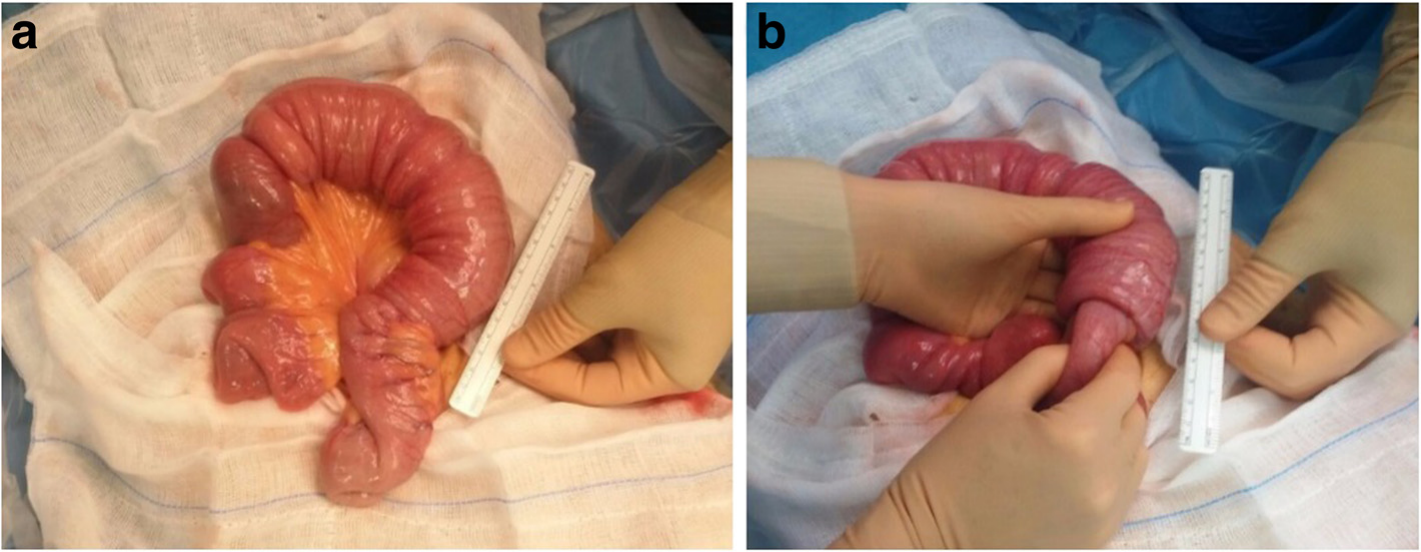
A thickened, congested, and inflamed ileoileal intussusception involving approximately 30 cm of intestine was found intraoperatively (**a**). A cautious manual reduction was performed (**b**)

He was then transferred to our Department of General Surgery to undergo an exploratory laparotomy in an emergency regime. The surgical exploration confirmed the intestinal intussusception involving approximately 30 cm of ileum. A cautious manual reduction of the intussusception was carried out, not showing signs of ischemia (Fig. 3). This procedure revealed a mass within the invaginated intestinal lumen, evocative for the lead point of the intussusception. The resection of approximately 15 cm of small bowel including the lesion was then performed. The restoration of bowel continuity was carried out with a manual side-to-side anastomosis.

**Fig. 4:**
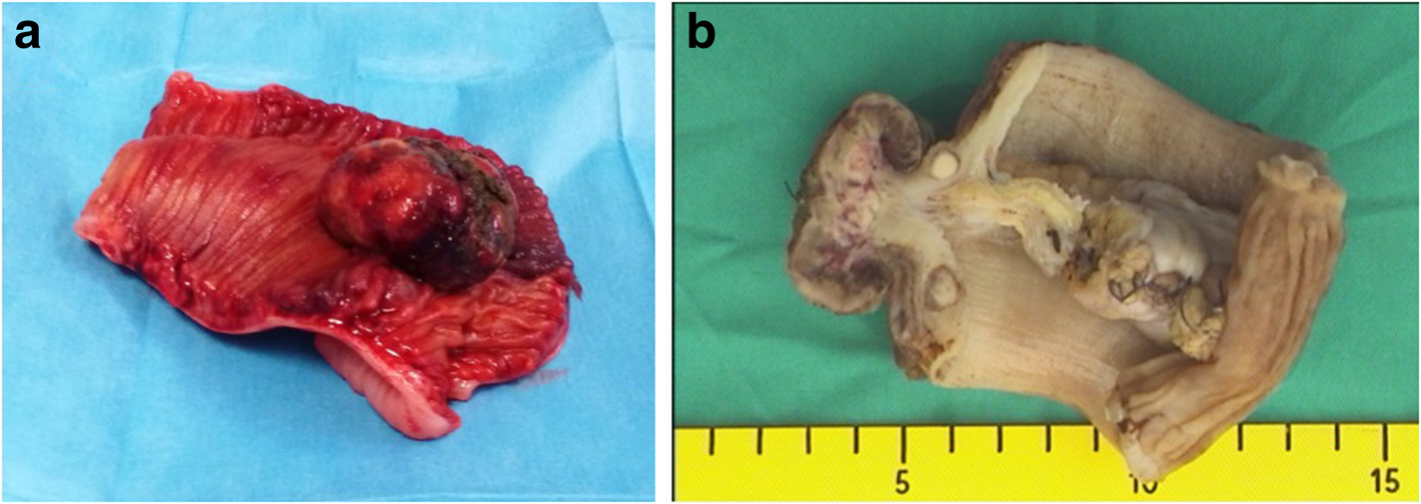
The surgical specimen after the resection of approximately 15 cm of the involved ileum (**a**). The lead point of the intussusception was an ulcerated polypoid lesion (**b**)

**Fig. 5:**
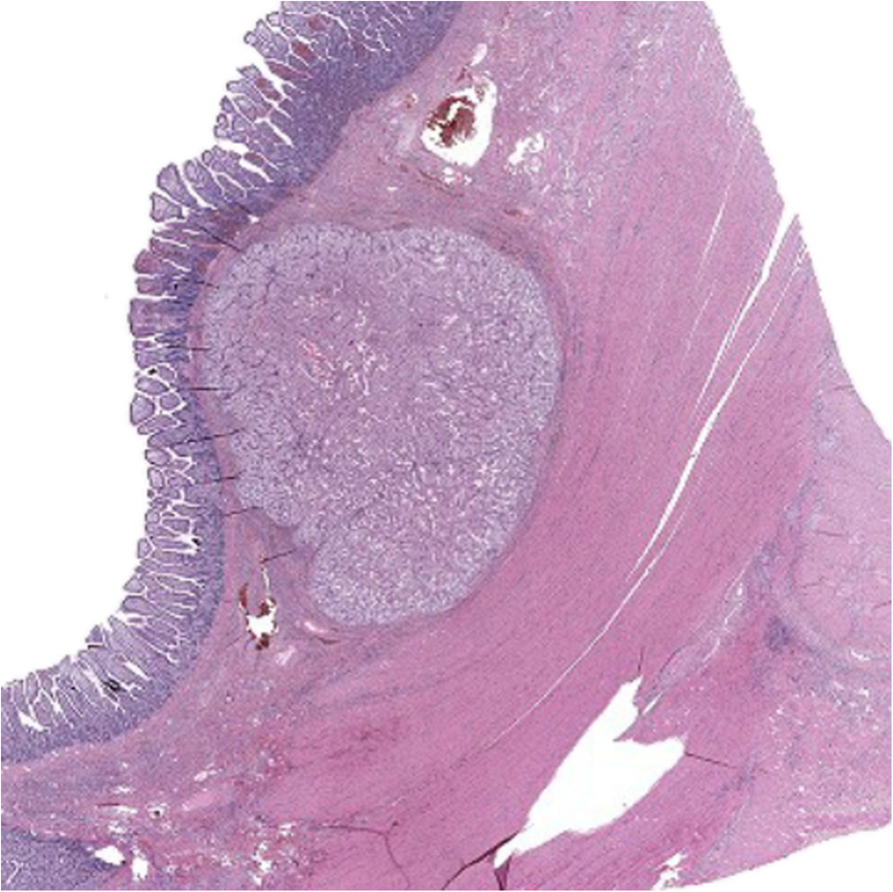
A histopathological examination of the ulcerated polypoid lesion demonstrated the presence of metastatic clear cells in the intestinal wall

Evaluation of the surgical specimen confirmed the presence of a voluminous polypoid lesion (4.5×4.5×3 cm) with surface ulceration (Fig. 4). A histopathological examination revealed that the mass was a clear cell carcinoma with immunohistochemical positivity for CD10 and focal positivity for vimentin (Fig. 5). This finding was suggestive for a clear cell RCC metastasis.

His postoperative course was characterized by pulmonary consolidation treated with antibiotics. He was discharged on the 22nd postoperative day. Two months after discharge he underwent a chest and abdominal CT scan that showed some “target-shaped” nodules in his liver and multiple enlarged lymph nodes. As a consequence of the CT scan findings, our oncologists decided to start a therapy with sunitinib.

## Discussion

Small bowel malignancies are rare and usually discovered because of their clinical manifestation [8]. Small bowel metastases represent only 10 % of all small bowel malignancies. Only 2 % of all tumors metastasize to the small intestine [9]. Common cancers metastasizing to the small bowel are from the lung, head and neck, breast, esophagus, and malignant melanomas [10]. Intestinal metastases from RCC represent only 7.1 % of all bowel metastases [9].

It is possible to diagnose intestinal intussusception with many diagnostic tools, such as abdominal US, plain abdominal films, upper GI contrast series, or barium enema examination. However, the most useful radiologic method to confirm intussusception is abdominal CT scan, with a reported diagnostic accuracy and sensitivity that may reach 100 % [3, 4, 11, 12]. Moreover, an abdominal contrast-enhanced CT scan provides information about the length and the localization of the GI tract involved, vascular impairment, and it may help staging the patient with suspected malignancy [12]. An abdominal CT scan is also able to distinguish an intussusception with a lead point (signs of bowel obstruction, bowel wall edema with loss of the classic three-layer appearance due to impaired mesenteric circulation, and demonstration of the lead mass) from that without a lead point (no signs of proximal bowel obstruction, target-like or sausage-shaped mass, or layering effect) [1, 2].

Although there are many diagnostic tools to diagnose intussusception, the diagnosis of intestinal invagination is often carried out on the operating table [1, 6, 7]. Surgery is the treatment of choice for bowel intussusceptions in adults. It still remains controversial whether to proceed with oncologic or limited resection and whether to reduce or not the intussusception [1, 4, 6, 7]. The best treatment for RCC metastases is the complete resection of the lesions [13].

RCC is the most frequent malignancy of the kidney, representing approximately 90 % of all solid renal tumors, and in 80 to 90 % of cases it appears to be a clear cells type. This neoplasia in 30 to 70 % of cases is diagnosed at an already advanced stage with local infiltration or distant metastases [14]. RCC may metastasize almost anywhere in the body and the most frequent sites of metastasis include the lymph nodes, lungs, liver, bones, brain, adrenal glands, and the contralateral kidney [15, 16]. More than one organ system is often involved in the metastatic process [15, 17].

Small bowel metastases from RCC are extremely rare, with an incidence that ranges from 0.7 to 14.6 % [15]. Diagnosis of GI metastatic lesions from RCC is often delayed, because these lesions are usually discovered as a result of their clinical presentation. The clinical presentation of GI metastases from RCC is usually GI bleeding due to the invasion of the intestinal vessels, or intestinal obstruction, or from the mass effect of the metastasis [15].

Viadana *et al.* [18] explained that hematogenous metastasization of RCC firstly involves the lung, through the renal vein and the inferior vena cava. From there, metastases may spread anywhere in the body. This hypothesis is supported by the fact that lungs are the most common site of metastasization from RCC [18]. Many renal masses remain asymptomatic for a long time and most of them are detected incidentally by the frequent use of imaging examinations for unrelated symptoms or diseases [19].

RCC has a low survival rate and patients with stage IV have a 5-year survival rate of approximately 8.2 % [20]. In this case, our patient is still alive more than 15 years after the original diagnosis, despite his development of metastases within both adrenal glands, brain, and small bowel.

Radiotherapy can be used for selected symptomatic patients with non-resectable brain or osseous lesions who do not respond to systemic treatment approaches. The use of targeting agents (tyrosine kinase inhibitors and immunotherapy) in patients with metastatic RCC led to a substantial improvement in progression-free and overall survival [19]. In this case, radiotherapy was used as the first-line treatment for the brain metastases, even if those lesions were not symptomatic, and a complete response was achieved.

Small bowel intussusception due to RCC metastasis is an exceptional event. Roviello *et al.* [15] presented only eight cases of small bowel metastases causing intussusception between 1939 and 2006 from a total of 21 case reports about GI metastases from RCCs (38.1 %). Their review showed that affected patients were mainly males, with a mean age of 62 years. They reported the jejunum as the most common location of the metastatic lesions, with an average interval to presentation from the primary tumor of 5 to 6 years. We managed to find in the literature another eight case reports about intussusception from RCC metastasis, seven of which were published during the last 10 years [6, 10, 16, 21–25]. The literature described that most intussusceptions from RCC metastasis occurred metachronously [10]. Among the eight cases reported by Roviello *et al.* [15], there are two in which intussusception appeared before the diagnosis of the primary RCC and one in which it appeared synchronically. Moreover, both Mishra *et al.* [10] and Hegde *et al.* [21] described cases in which the clinical manifestation of the metastasis enabled the primitive RCC to be diagnosed.

Kassouf *et al.* [26] reported that in the literature, abdominal metastasis was found approximately 97 months after nephrectomy for pT1 RCC and approximately 79 months after pT3 RCC. Our patient was metastatic at the beginning; therefore, the risk of relapse was very different from patients without metastases. The peculiarity of our case is the long disease-free period (approximately 10 years) between the successful treatment of the brain metastasis and the small bowel relapse.

Follow-up intensity and duration in patients operated for RCC is still nowadays controversial. Many surveillance programs exist, which are based on different scores to evaluate the risk of recurrence. According to the 2010 update of the European Association of Urology guidelines on RCC, to monitor correctly patients after surgery it is fundamental to create a surveillance algorithm recognizing both the patient risk profile and the efficacy of the treatment [19]. Therefore, patients affected by RCC need individualization in the setting of an optimized follow-up.

## Conclusions

Unexplained intestinal subocclusion presenting with vomiting and anemia is a very aspecific clinical manifestation that could be derived from many pathological entities. In a patient with a history of RCC and without other evident causes, the possibility of a small bowel intussusception due to a RCC metastasis should be taken into account.

## Abbreviations

CT, computed tomography; GI, gastrointestinal; RCC, renal cell carcinoma; US, ultrasonography
